# Short treatment of peripheral blood cells product with Fas ligand using closed automated cell processing system significantly reduces immune cell reactivity of the graft in vitro and in vivo

**DOI:** 10.1038/s41409-022-01698-3

**Published:** 2022-05-10

**Authors:** Galina Rodionov, Michal Rosenzwaig, Michal Schrift Tzadok, Moran Kvint, Elazar Gevir, Elina Zorde-Khvalevsky, Amnon Peled, Shai Yarkoni, Amos Ofer

**Affiliations:** 1 Cellect Biotherapeutics Ltd, Kfar-Saba, Israel; 2 Goldyne Savad Institute of Gene Therapy, Hebrew University of Jerusalem, Jerusalem, Israel

**Keywords:** Haematological cancer, Haematopoietic stem cells, Bone marrow transplantation, Apoptosis, Cell death and immune response

## Abstract

Mobilized peripheral blood cells (MPBCs) graft and peripheral blood cells apheresis are used for bone marrow transplantation and for treatment of graft versus host disease (GvHD). We demonstrate that a short treatment of MPBCs with Fas ligand (FasL, CD95L) for 2 h using a closed automated cell processing system selectively induces apoptosis of specific donor T cells, B cells and antigen presenting cells, but, critically, not CD34^+^ hematopoietic stem cells and progenitors, all of which may contribute to an increased likelihood of graft survival and functionality and reduced GvHD. Treated cells secreted lower levels of interferon-gamma as compared with control, untreated, cells. Moreover, FasL treatment of immune cells increased signals, which led to their phagocytosis by activated macrophages. FasL treated immune cells also reduced the ability of activated macrophages to secrete pro-inflammatory cytokines. Most importantly, FasL ex vivo treated MPBCs prior to transplantation in NOD-SCID NSG mice prevented GvHD and improved stem cell transplantation in vivo. In conclusion, MPBCs, as well as other blood cell products, treated with FasL by automated manufacturing (AM), may be used as potential treatments for conditions where the immune system is over-responding to both self and non-self-antigens.

## Abstract (web summary)

A brief, ex vivo incubation of human G-CSF-mobilized peripheral blood grafts with multimerized Fas-Ligand (CD95L), using a closed automated cell processing system, significantly reduces immunoreactivity of the graft in vitro and in vivo.

## Introduction

Allogeneic hematopoietic stem-cell transplantation (HSCT) has been used to cure a wide range of malignant and non-malignant hematological diseases by replacing host cells that contribute to, or are causative of, various disease states. Following transplantation of granulocyte-colony stimulating factor (G-CSF)-mobilized peripheral blood cells (MPBCs) [1], T cells contained in the graft promote hematopoietic engraftment, T-cell immunity, and potent graft versus tumor effects (GvT) [2–4]. A double-edged sword, these same T-cells can also mediate GvHD when they recognize and respond to host allo-antigens presented by host or donor antigen-presenting cells (APCs) [5, 6]. A variety of in vivo or ex vivo modalities are available to prevent GvHD, but these same agents and procedures attenuate engraftment and long-term chimerism, immune reconstitution, increase the risk of infection and/or abrogate essential T-cell-mediated GvT effects [7, 8]. This is because of the morphological similarity of the GvHD causing cells and the cells that support engraftment and GvT. In our previous study, we have shown that brief incubation of human stem cells grafts with hexameric Fas ligand (FasL) selectively induces apoptosis of specific donor T-cell subsets and APCs but not of CD34^+^ cells. This effect uncouples GvHD and GvT, without attenuating the engraftment potential of the cells in vivo. We have shown that ex vivo FasL treatment of the graft attenuates the detrimental immune response of the graft against host tissue [9].

It has been suggested that the administration of ex vivo generated, tolerogenic immune cell populations could provide a tractable therapeutic strategy for autoimmunity. Current management of autoimmunity involves administration of systemic immunosuppressive drugs coupled with other interventions such as anti-inflammatory therapies.

Based on numerous pre-clinical studies, there is growing evidence of the potential benefits of cell-based therapies for the treatment of sepsis and ARDS. Several cell types have been recently used for the treatment of both syndromes showing variable efficiency [10]. Embryonic stem cells (ESC), multipotent stem (or stromal) cells and epithelial progenitor’s cells (EpPC) have been used for both diseases. In recent decades, new therapeutic strategies have been developed; nevertheless, none has proven effective [11]. Many of these therapies have employed the use of immunosuppressive regimens [12].

In this study, we demonstrated that short treatment of peripheral blood cells apheresis with FasL, using closed automated cell processing system, produce a cell composition which reduced immune cell reactivity in vitro and in vivo and can be potentially used to treat rejection (including GvHD), ARDS and sepsis.

## Materials and methods

### Apheresis sample collection and FasL treatments

G-CSF mobilized peripheral blood cells (MPBCs) were collected by apheresis from healthy donors at the Sheba Medical Center, (Institutional Review Board [IRB] approval Numbers SMC-5179-18 and SMC-7440-20.), Ezer Mezion (IRB approval No SMC-5199-18) or at Rambam Medical Center, (IRB approval No. RMB-0210-20).

Ex vivo incubation of MPBCs with FasL, was performed in a closed infusion bag. In brief, the plasma of MPBCs was exchanged with incubation medium (SCGM, CellGenix, Portsmouth, NH, USA) using the Fresenius Kabi LOVO functionally closed automated cell processing system, 100 ng/ml or 400 ng/ml of hexameric FasL (MegaFasL, AG-40B-0130-C010, Adipogen, San Diego, CA, USA, or FasCELLECT, FP00001, SBC) was added to cell suspension. After 2 h at 37 °C in 5% CO_2_, the incubation medium was exchanged with transplantation buffer composed of PlasmaLyte A (FKE0324, Baxter Healthcare, Norfolk, UK) and 5% Human Albumin Solution (Zenalb20, Bio Products Laboratory Limited, Hertfordshire, UK) using the Fresenius Kabi LOVO functionally closed automated cell processing system. Untreated control MPBCs were stored at 2–8 °C until use.

### CD34^+^ purification and expansion

CD34^+^ cells were purified from untreated and FasL treated human MPBCs using CD34^+^ cell separation microbeads (Miltenyi, Cat. No 130-046-703). More than 85% CD34^+^ cells with more than 80% viability were detected on Day 0.

For direct exposure with FasL, CD34^+^ cells were purified from untreated MPBCs and were frozen immediately following purification and maintained in the liquid nitrogen. Cells were thawed on the day of the experiment, with more than 85% CD34^+^ cells and more than 80% viability were detected after thawing. Cells were resuspended in complete RPMI at a concentration of 0.5 × 10^6^ cells/ml and incubated in the absence or presence of 100 ng/ml FasL protein for 5 min, 1 h and 4 h, followed by two washing steps in PBS. The homogeneity of plate seeding (Day 0) was tested using CellTiter-Glo® 2.0 cell viability assay (Promega, G9241).

Cells were resuspended in StemSpan™ SFEM II medium (Stemcell, 09605) containing StemSpan™ CD34^+^ Expansion Supplement (Stemcell, 02691) and seeded into 48-wells plate, 5000 cells/well (according to Stemcell recommendations) for 7-days expansion.

Cell number, viability (trypan blue exclusion test) and %CD34^+^ cells (FACS) were tested on Day 7. More than 85% cell viability was detected in the expanded cultures and total cell number per well was normalized according to CellTiter-Glo seeding data on Day 0.

The percentage of CD34^+^ cells was tested on Day 7 using FACS analysis.

The percentage of multi-potent progenitors (CD38^neg/low^CD90^−^CD45RA^−^), self-renewal hematopoietic stem cells (HSCs, CD38^neg/low^CD90^+^CD45RA^−^), lymphoid-primed multipotent Progenitors (LMPPs, CD38^neg/low^CD90^−^CD45RA^+^), oligo-potent progenitors (OPPs, CD38^high^) [13], early-stage megakaryocyte progenitors (MkPs) (CD38^neg/low^CD41a^+^) and more mature MkPs (CD38^high^CD41a^+^) [14–16].

### Glutathione (GSH) level

Untreated MPBCs and MPBCs treated with 100 ng/ml FasL for 2 h were stained with FreSHTracer (FT) to evaluate GSH level on CD34^+^ cells according to Jeong et. al. [17] FT, a fluorescent dye that, in living cells, can penetrate the membrane and bind GSH has a different absorption and emission spectra when bound to reduced glutathione (510 nm) and oxidized glutathione (580 nm). The F510/F580 ratio (FR) correlates with GSH levels and redox potential [17].

### Flow cytometry

MPBCs or mouse bone marrow cell (BMC) suspensions were lysed using Ammonium–Chloride–Potassium (ACK) Lysis buffer (A10492-01, Thermo Fisher Scientific, Waltham MA, USA) and 1 × 10^6^ cells were stained with various combinations of fluorescence- conjugated anti-human antibodies (except for anti-CD45 antibody that was of either mouse or human origin as indicated in the text). For cell surface staining, cells were incubated with antibodies for 15 min at RT or at 4 °C in the dark.

The following antibodies from BD Biosciences US were used: anti-human CD45 (340910), anti-human CD34 (345802), anti-human CD38 (646851), anti-human CD41a 555466, anti-human CD3 (345765).

The following antibodies from Miltenyi Biotech, Bergisch Gladbach, Germany were used: anti-human CD90 (130-114-862), anti-human CD45RA (130-117-744) anti-human CD45 (130-110-637), anti-human CD3 (130-113-138), anti-human CD19 (130-113-646), anti-human CD33 (130-111-021), anti-human CD25 (130-113-284), anti-human HLA-DR (130-111-792), anti-mouse CD45 (130-110-665), anti-human CD34 (130-113-176), tandem signal enhancer (130-099-888), Fc blocker mouse (130-092-575); matched isotype controls were used as negative control.

Induction of apoptosis was evaluated using Annexin V and 7-Aminoactinomycin D (7-AAD) (BMS500FI and 00-6993 respectively, Invitrogen, Thermo Fisher Scientific, Waltham MA, USA) staining. Early apoptosis was calculated as % AnnexinV^+^/7-AAD^−^ cells of indicated populations.

Data was acquired using MACSQuant analyzer 10 and analyzed using MACSQuant software (Miltenyi Biotech, Bergisch Gladbach, Germany). Analysis for CD34^+^ cells was conducted according to the International Society of Hematotherapy and Graft Engineering (ISHAGE) guidelines [18, 19].

### IFN-γ measuring

Untreated and FasL AM treated MPBCs were cultured in complete RPMI supplemented with 30 ng/ml of anti-human CD3 antibody (16-0037-81, eBioscience) and 1000 IU/ml IL-2 (200IL-010, R&D systems) for specific T-cell activation. Concentration of IFN-γ secreted to the medium was measured 24-hrs post-stimulation using ELISA (DIF50, R&D systems) and normalized per seeded CD3^+^ cell number in culture, which was calculated using results of cells count using the trypan blue exclusion test and %CD3 cells evaluated by FACS analysis.

### Xenogeneic GvHD and engraftment models

Non-obese diabetic-severe combined immune deficiency (NOD-SCID) IL2Rγnull (NSG) mice (Jackson Laboratory, Bar Harbor MN, USA) were housed in a pathogen-free facility and handled in accordance with the guidelines of the Animal Care and Use Committee (ACUC) of the Rabin Medical Center, Petach, Tikva, Israel. For each experiment female NSG mice (7–9 weeks) were randomized according to age and body weight. The mice were irradiated with 2 or 2.75 Gy (CLINAC-DBX linear accelerators, Varian Medical System, Palo Alto, CA, USA) 24–48 h prior to intravenous infusion of ∼5 × 10^6^ unfractionated MPBCs (total nucleated cell (TNC), FasL AM treated MPBCs or vehicle control containing the test material diluent, 8–10 mice per group were transplanted (acceptable group size based on statistical and ethical considerations). The grafts administered to each group contained an equivalent number of CD34^+^ cells (Supplementary Table 2). Monitoring of GvHD clinical score was carried out twice weekly according to the murine clinical grading system described by Cooke et al. [20] Weight loss, hunched posture, skin lesions, dull fur, and mobility were each assigned scores of 0 (absent), 1 (moderate) or 2 (severe). The investigator was blinded to the group allocation during each experiment.

Mice were sacrificed in case of weight loss ≥20% of initial weight or upon reaching a clinical GvHD score of ≥7. NOD.Cg-Prkdcscid IL-2rgtm1Wjl/SzJ (NSG) mice are a valuable tool for studying Graft-versus-Host-Disease (GvHD) induced by human immune cells. It was already reported by others [21] that induction of GvHD in these mice induced robust damage to the bone marrow associate with fever cytopenia, apoptotic crypt cells in the gut, and liver damage. All the observed effect are signs of acute GvHD.

From the first time point of scarification of mice onwards, the mean clinical score is not presented due to the small number of mice with a relatively low clinical score remaining in the test groups, which leads to high variability and makes the analysis inaccurate.

At each of studies termination endpoint, BM were harvested, the BMC were extracted, and human cell engraftment was analyzed by flow cytometry.

### Colony Forming Unit assay

For detection of human colony forming cells from mouse BM, single cell suspensions of mouse BM were lysed using ACK Lysis buffer (A10492-01, Thermo Fisher Scientific, Waltham MA, USA), diluted with IMDM + 2% FBS and MethoCult™ H4534 medium (StemCell technologies, Vancouver, BC, Canada). CD34^+^ cells amount in total BMC was evaluated using cell count by trypan blue exclusion test and FACS analysis. BMC were seeded in a 6 well SmartDish plate, 150 CD34^+^ cells/well (all reagents were purchased from StemCell Technologies, Vancouver, BC, Canada) and incubated at 37 °C, in 5% CO_2_, with ≥95% humidity for 14 days. The number of colonies was expressed per 100 seeded CD34^+^ cells.

### Phagocytosis assay

Untreated and FasL AM treated MPBCs were resuspended at 10^6^ cells/ml in 25 ml of completed RPMI and incubated overnight at 37 °C, 5% CO_2_.

Macrophages were purified from peripheral blood mononuclear cells (PBMCs) and incubated for 7 days with 50 ng/ml GM-CSF (PEPROTECH, 300-03) and M-CSF (PEPROTECH, 300-25). Tested cells were stained with CFSE (eBioscience, 65-0850-84) per manufacturer’s protocol and co-incubated with macrophages at noted ratios for 3.5 h. All cells were stained with anti-human CD11b antibody (BioLegend, 301310) to define macrophages.

Percentage of phagocytic (CFSE^high^) activated macrophages per total CD11b^+^ population was analyzed by FACS analysis [22]. CFSE-stained macrophages of MPBCs were excluded from FACS analysis as described in a gating strategy in Supplementary Fig. 2.

Supernatants of macrophages incubated without cells, with untreated or FasL AM treated MPBCs, and cells incubated without macrophages were collected following 3.5 h incubation and stored at −80 °C until testing. Supernatants were tested using Quantikine ELISA kit Human interluekin-8 (IL-8), interluekin-6 (IL-6) and tumor necrosis factor-alpha TNFα (R&D Systems, D800C, D6050 and DTA00D respectively) according to manufacturer instructions.

### Statistical analysis

Statistical tests are justified as appropriate for each figure.

Two-tailed paired *T*-test for comparison MPBCs vs MPBCs + FasL AM samples, data are presented as average of 3 technical replicates for each, of at least 3 independent experiments, each performed using individual MPBCs donation.

Multiple unpaired *T* tests, Mann–Whitney test, and Log-rank (Mantel–Cox) test for animal studies, at least 8 animals/ group was tested.

In case the representative experiment is presented, multiple unpaired Student’s *T* test was applied for technical replicates of individual representative tests.

The data meet the assumptions of the tests, the variation is estimated within each group of data, the variance between the groups that are being statistically compared was found similar.

## Results

### Short treatment of MPBCs with Fas ligand, using closed automated cell processing system, results in selective reduction of CD3^+^ T cells while maintaining CD34^+^ viability and functionality

**Fig. 1:**
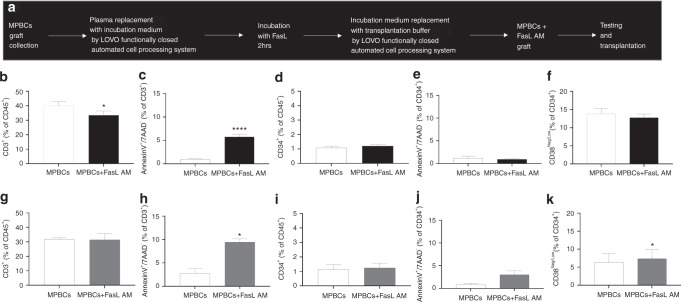
FasL AM MPBCs treatment significantly reduces CD3^+^ cells without compromising the quantity and quality of CD34^+^ cells. **a** FasL AM MPBCs treatment process performed using the Fresenius Kabi LOVO functionally closed automated cell processing system, designed for HSCT. MPBCs graft characterization following (**b**–**f**) 100 ng/ml FasL AM treatment or (**g**–**k**) 400 ng/ml FasL AM treatment. Percentage of (**b**, **g**) CD3^+^ and (**d**, **i**) CD34^+^ cells per total CD45^+^ population. Percentage of annexin V positive (**c**, **h**) CD3^+^ and (**e**, **j**) CD34^+^ cells per 7AAD^−^ population. **f**, **k** Percentage of multi potent stem and progenitor cells (CD34^+^CD38^neg/low^) per total CD34^+^7AAD^−^ population. **b**–**e**: *N* = 16, **f**: *N* = 9, **g**–**k**: *N* = 3. Mean ± SEM, *P ≤ 0.05, **P ≤ 0.01, ***P ≤ 0.001; Paired *T*-test.

The FasL AM treatment of MPBCs using the Fresenius Kabi LOVO functionally closed automated cell processing system was developed to replace the buffer exchange steps by centrifugation in the manual process (Fig. 1a). MPBCs from 19 healthy donors were separately incubated for 2 h with 100 ng (*N* = 16) or 400 ng/ml (*N* = 3) hexameric FasL. FasL AM treatment significantly reduces the percentage of lymphocytes and, accordingly, increases the percentage of granulocytes in the MPBCs without significantly affecting macrophages (Supplementary Fig 1). The absolute number TNC, CD34^+^, and the percentage of immune subpopulations before and after FasL treatment are presented in Supplementary Table 1.

**Fig. 2:**
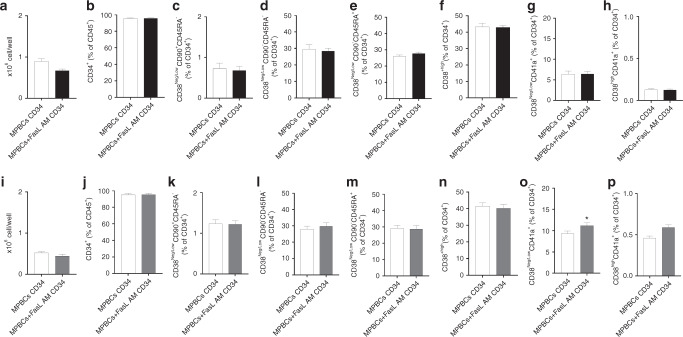
FasL AM MPBCs treatment does not affect the expansion capacity of CD34^+^ cells. Characterization of HSPCs purified from 100 ng/ml FasL AM treated (**a**–**h**) or 400 ng/ml FasL AM treated (**i**–**p**) MPBCs and from control MPBCs followed by 7 days expansion. Total cell number per well, (**b**, **j**) percent of CD34^+^ cells per total cells in culture. Percentage of HSPCs subpopulations per total CD34^+^ population: (**c**, **k**) self-renewal HSCs (CD34^+^CD38^neg/low^ CD90^+^CD45RA^−^), (**d**, **l**) MPPs (CD34^+^CD38^neg/low^ CD90^−^CD45RA^−^), (**e**, **m**) LMPPs (CD38^Neg/Low^CD90^−^CD45RA^+^), (**f**, **n**) OPPs (CD38^+high^) (**g**, **o**) early-stage MkPs (**h**, **p**) and more mature MkPs (CD38^neg/low^CD41a^+^ and CD38^high^CD41a^+^, respectively). *N* = 3, Mean ± SEM, *P ≤ 0.05 Paired *T*-test.

Early apoptosis signals and significant reductions in the percentage of CD3^+^ T cells were detected in the FasL-treated samples while CD34^+^ percentage and viability were unaffected (Fig. 1b–e, g–j). MPBCs AM treatment with 100 ng/ml FasL did not affect and with 400 ng/ml FasL significantly increase the percentage of immature CD34^+^CD38^low^ stem cells (Fig. 1f, k). Furthermore, 100 ng/ml as well as 400 ng/ml FasL treatment did not reduce the expansion capacity of CD34^+^ cells purified from FasL AM treated MPBCs vs. CD34^+^ purified from untreated MPBCs (Fig. 2a, b, i, j). No differences were observed in the percentage of HSPCs self-renewal HSCs (CD34^+^CD38^neg/low^CD90^+^CD45RA^−^, Fig. 2c, k), multipotent progenitors (MPPs, CD34^+^CD38^neg/low^CD90^−^CD45RA^−^, Fig. 2d, l), LMPPs (CD38^neg/low^CD90^−^CD45RA^+^, Fig. 2e, m), OPPs (CD38^high^, Fig. 2f, n), early-stage MkPs, (CD38^neg/low^CD41a^+^) and more mature MkPs (CD38^high^CD41a^+^) (Fig. 2g, h, o, p). Interestingly, a significant increase in the percent of early-stage MkPs was found when CD34^+^ cells purified from MPBCs were incubated with 400 ng/ml FasL for 7 days (Fig. 2o).

These results suggest a selective effect of the FasL treatment on CD3^+^ T cells, with a preservation of CD34^+^ progenitor cells viability and expansion potential.

### FasL AM treatment of MPBCs decreases B cells, myeloid cells and activated T cells populations, reduce the antigen presentation potential, and attenuates IFN-γ secretion

**Fig. 3:**
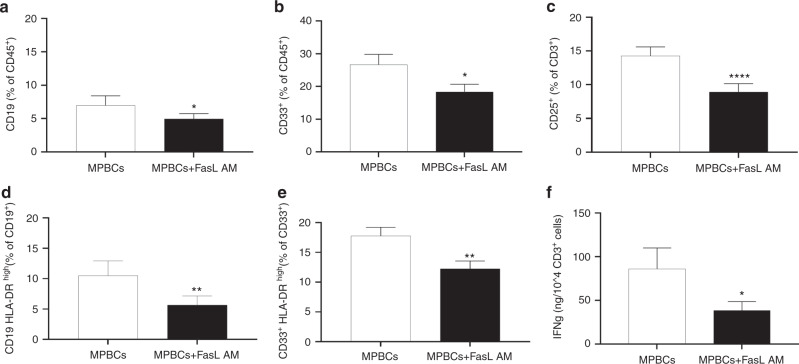
FasL AM treatment of MPBCs decreases B cells, myeloid cells and activated T cells populations, reducing the antigen presentation potential and T cell activation potential. MPBCs graft characterization following 100 ng/ml FasL AM treatment: (**a**) Percentage of B-lymphocytes (CD19^+^), (**b**) Myeloid cells (CD33^+^) and (**c**) Activated T cells (CD25^+^) per total CD45^+^ population. The percentage of HLA-DR^high^ expressing on B-lymphocytes (**d**) and on myeloid cells (**e**) as marker for antigen presentation potential per total CD19^+^ or CD33^+^ population. *N* = 7, *P ≤ 0.05, **P ≤ 0.01, ****P ≤ 0.0001; Paired *T*-test. **f** Concentration of IFN-γ secreted to the medium, measured 24 h post T cell specific and normalized per seeded CD3^+^ cell number in culture. *N* = 8, *P ≤ 0.05; Paired *T*-test.

Ex vivo FasL AM treatment of MPBCs significantly reduces the percentage of B (CD19^+^) cell and myeloid (CD33^+^) cell subsets (Fig. 3a, b) and the percentage of HLA-DR^high^ expressing B-lymphocytes and myeloid cells (Fig. 3d, e). FasL AM treatment also significantly reduced the percentage of activated T-lymphocytes (CD3^+^CD25^+^, Fig. 3c). We have previously shown the effect of FasL on different subsets of  T  cells including Naïve (CCR7^+^ CD45RA^+^CD95^−^LFA1^low^), T_SCM_ (CCR7^+^CD45RA^+^CD95^+^LFA1^high^), central memory (CM, CCR7^+^CD45RA^−^), effector memory (EM, CCR7^−^CD45RA^−^), effector (eff, CCR7^−^CD45RA^+^) as well as TH1, (CD4^+^CXCR3^+^), TH17 (CD4^+^CCR6^+^CXCR3^−^) and TC1 (CD8^+^ CXCR3^+^). Fas (CD95^+^) expressing T-cell populations shows correlation with FasL induced apoptosis of this cells. We found that CD8scm are expressing high levels of CD95 and respond strongly to FasL induction of apoptosis*,* furthermore we have shown that FasL treatment did not affect Regulatory CD4^+^ and CD8^+^ T cells (CD25^+^FoxP3^+^) % out of total CD3 cells [9]. In additional we have found that the relative presence of T regulatory (CD25^+^CD127^+low^ expressing cells) is actually increases in activated T helper (CD4^+^CD25^+^) population of MPBCs following FasL treatment (Supplementary Fig 3).

In order to mimic the allo-activation that occurs following infusion of a stem cells graft, MPBCs or FasL AM treated MPBCs were exposed to anti-human CD3 antibody and IL-2 for 24 h and the level of IFN-γ was tested. FasL AM treatment of MPBCs significantly reduces the secretion IFN-γ in the culture (Fig. 3f).

### FasL AM treatment of MPBCs increases their phagocytosis and reduces pro-inflammatory cytokine secretion by activated macrophages

**Fig. 4:**
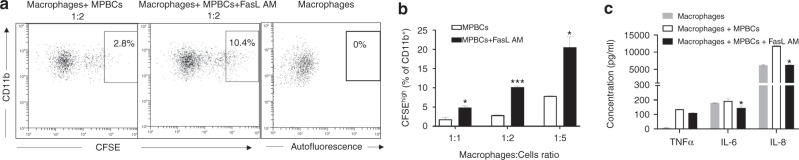
FasL AM treatment of MPBCs increases their phagocytosis and reduces proinflammatory cytokine secretion by activated macrophages. 100 ng/ml FasL AM treated and non-treated MPBCs were incubated with activated macrophages at different ratios. Percentage of high phagocytic (CFSE^+high^) macrophages per total CD11b^+^ population as well as secretion of TNFα, IL-6 and IL-8 were evaluated. Percentage of high phagocytic (CFSE^+high^) macrophages: (**a**) Representative flow cytometry dot plots, (**b**) results of the representative experiment are presented as mean ± SEM of technical duplicates, *P ≤ 0.05; ***P ≤ 0.001, Multiple unpaired *T*-test. **c** The effect of engulfment of apoptotic cells by activated macrophages on TNFα, IL-8 and IL-6 secretion. Results of a representative experiment are presented as Mean ± SEM of technical duplicates. Macrophages: cell ratio 1:5; *P ≤ 0.05; Multiple unpaired *T*-test.

Apoptosis or programmed cell death is essential for morphogenesis, cell homeostasis, injury repair, immune tolerance, and resolution of inflammation in multicellular organisms [23–25]. The clearance of apoptotic cells is a highly regulated mechanism, normally associated with anti-inflammatory response [26, 27]. Signals that control phagocytosis by macrophages (eat-me signals) are the key to the recognition of extracellular cargos and the initiation of the phagocytosis process are convenient targets for therapeutic modulation as typically occurs with phagocytosis of opsonized pathogens through complement. The uptake of apoptotic cells by macrophages is known to potentially amplify and preserve an anti-inflammatory state [28–30] FasL AM treated and non-treated MPBCs were incubated with activated macrophages at different ratios. FasL AM treatment significantly increases the percentage of phagocytic (CFSE^high^) macrophages per total CD11b^+^ population (Fig. 4a, b). Furthermore, FasL AM treated immune cells significantly reduced IL-8 and IL-6 cytokine secretion and TNF-α from activated macrophages (non-significant, Fig. 4c).

### FasL AM treatment of MPBCs attenuates acute GvHD in a xenogeneic GvHD model, enhances the short-term engraftment of human cells and does not impair re-constitution potential of HSPC in mice 4 weeks post-administration

**Fig. 5:**
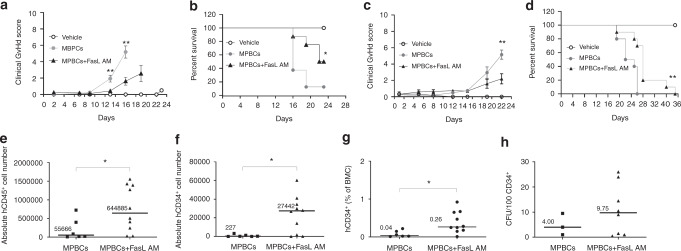
FasL AM treatment of MPBCs attenuates acute GvHD in a xenogeneic GvHD model, enhances the short-term engraftment of human cells and does not impair re-constitution potential of HSPC in mice 4 weeks post administration. GvHD score (**a**, **c**) and percent survival (**b**, **d**) of two independent experiments in γ-irradiated NSG mice transplanted with either 100 ng/ml FasL AM treated or untreated MPBCs control grafts. **a**, **b** Experiment 1: MPBCs + FasL AM and MPBCs *N* = 8 mice/group, Vehicle *N* = 2 mice/group; **c**, **d** Experiment 2: MPBCs + FasL AM and MPBCs *N* = 10 mice/group, Vehicle *N* = 3 mice/group; Mean ± SEM, Multiple *T* tests; Kaplan–Maier survival curve, Log-rank (Mantel–Cox) test; *P ≤ 0.05, **P ≤ 0.01. **e**–**h** Engraftment of human cells in the bone marrow of treated mice, 4 weeks post-transplantation. The absolute cell numbers of (**e**) human CD45^+^and (**f**) human CD34^+^, (**g**) percentage of CD34^+^ per total BM cells (BM) population; MPBCs+FasL AM *N* = 10, MPBCs *N* = 6. **h** CFU capacity of BMC derived from mice and cultured for 2 weeks for colony growth. MPBCs + FasL AM *N* = 8, MPBCs *N* = 3. **e**–**h** Each data point represents an individual mouse, horizontal lines represent the median of each treatment group *P ≤ 0.05, Mann–Whitney test.

We studied human MPBCs engraftment and the development of GvHD in γ-irradiated NSG mice transplanted with either 100 ng/ml FasL AM treated or control grafts (Fig. 5).

We found that the mean clinical GvHD score of control MPBCs infused mice was significantly higher than GvHD score of FasL AM treated MPBCs infused mice 13–24 days post-transplantation (Fig. 5a, c). GvHD progressed significantly more intensive in control-MPBCs mice. The first study (Fig. 5a, b) was terminated on day 24. At this time point, we found a statistical difference in percent survival of treated and untreated groups (Fig. 5b). In the second study, none of the mice of the control MPBCs group survived more than 24 days post-transplantation, by contrast, part of the mice transplanted with FasL AM-treated-MPBCs survived to day 40 following transplantation (Fig. 5d).

Engraftment of human CD45^+^ leukocytes and human CD34^+^ hematopoietic progenitor cells was significantly higher in the BM aspirates derived from FasL AM treated MPBCs compared to control (Fig. 5e–g). A non-statistically significant increase in the number of human colony forming units (CFU) was found in the BM aspirates derived from FasL treated MPBCs compared to control (Fig. 5h). These results demonstrate that ex vivo FasL AM treatment reduces GvHD and does not impair engraftment of FasL treated MPBCs progenitors.

Since FasL treatment of MPBCs is used to prevent GvHD in transplanted leukemic patients we focused our studies on the effect of FasL on graft versus leukemia (GvL). We already shown in our previous published paper that FasL induced depletion of T cells is selective. Not all T cell subpopulations are equally or completely depleted. Furthermore, we have shown in vivo that whereas depletion of T cells could reduce GvHD it did not inhibit or reduced GvL [9]. We further show that FasL treatment does not affect the ability of activated T cells to proliferate and kill leukemic cells in vitro (Supplementary Fig 4).

### FasL treatment of CD34^+^ HSPCs increase their stemness

**Fig. 6:**
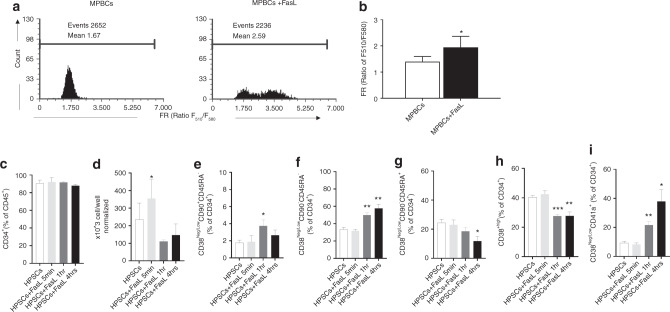
FasL treatment of CD34^+^ HSPCs increase their stemness. **a**, **b** FasL treatment enhances HSPCs stemness demonstrating higher F510/F580 fluorescence ratio (FR) correlated with Glutathione (GSH) level. (**a**) Representative flow cytometry histogram (**b**) results of 3 independent experiments of MPBCs treatment with 100 ng/ml of FasL; Mean ± SEM *P ≤ 0.05; Paired *T*-test. **c**–**i** 7 Days expansion capacity after short (5 min, 1 h and 4 h) exposure of purified CD34^+^ cells with 100 ng/ml FasL. (**c**) Percentage of CD34^+^ cells per total cell population, (**d**) total cell number per well, (**e**–**i**) percentage of HSPCs subpopulations per total CD34^+^ population: (**e**) Self-renewal HSCs (CD34^+^CD38^neg/low^CD90^+^CD45RA^−^), (**f**) MPP (CD34^+^CD38^neg/low^ CD90^−^CD45RA^−^), (**g**) LMPP (CD38^Neg/Low^CD90^−^CD45RA+), (**h**) OPP (CD38^+high^), (**i**) early-stage MkPs (CD38^Neg/low^CD41a^+^), (**c**–**h**): *N* = 3, (**i**) *N* = 2, Mean ± SEM, *P ≤ 0.05, **P ≤ 0.01, ***P ≤ 0.001 Paired *T*-test.

As demonstrated in Fig. 5e–h, engraftment of human CD34^+^ hematopoietic progenitor cells were higher in the BM aspirates derived from FasL treated MPBCs compared to control. A correlation between the stem cell potential (stemness) of the CD34^+^ HSPCs cells and GSH levels in the cells has been demonstrated [17]. GSH is an antioxidant found in millimolar levels in cells. Low levels of GSH indicate that the cell is under oxidative stress, detected by low oxidative stress resistance capacity (ORC), resulting in lower stemness potential of the cell [17]. Interestingly, we found that FasL treated MPBCs had an increased level of GSH compared to control MPBCs (Fig. 6a, b), as seen by the fluorescence ratio (FR) between reduced glutathione (GSH: 510 nm) and its oxidized form (580 nm). To further study the effect of FasL on the differentiation of CD34^+^ cells, CD34^+^ cells were purified from untreated MPBCs and MPBCs treated with 100 ng/ml FasL for a short time (5 min, 1 h and 4 h) followed by washing and seeding for 7 days culture in HSPCs expansion medium. The cell quantity after 7 days was between 20 to 100-fold vs. day 0 (data not shown) and the percent of CD34^+^ cells in expanded culture was 80–96% (Fig. 6c). These results show that the short exposure (5 min) of FasL significantly increase cell proliferation (Fig. 6d) but did not affect CD34^+^ cell composition after 7 days expansion (Fig. 6e–i). However, treatment of CD34^+^ cells with FasL for 1 h and 4 h prior to 7 days incubation did not affect the cell proliferation (Fig. 6d) but significantly increased the self-renewal of HSCs (CD34^+^CD38^−^CD90^+^CD45RA^−^, Fig. 6e), multi-potent progenitors (CD34^+^CD38^neg/low^CD90^−^CD45RA^−^, Fig. 6f) and early-stage MkPs (CD38^Neg/low^CD41a^+^, Fig. 6i). The significant reduction of more mature (LMPPs and OPPs) cells (Fig. 6g, h) are in correlation with an increase of the self-renewals population. These results suggest that FasL reduced oxidative stress and increased stemness properties in the cultures of MPBCs and reduced the differentiation potential of early HSPCs.

## Discussion

In this study, we have shown that brief incubation of human mobilized peripheral blood cells (MPBCs) graft with FasL using closed automated cell processing system selectively induces apoptosis of mature/activated immune cells but not of CD34^+^ hematopoietic stem cells and progenitors. We have also shown, for the first time, that FasL treatment of immune cells increase their eat me signals and led to their phagocytosis by activated macrophages. Furthermore, our results suggest that FasL treatment has the capacity to increase MPBCs-CD34^+^ cells self-renewal population in culture by reducing oxidative stress, or, under different conditions, induce their proliferation without affecting cell composition.

Glutathione plays a key role in maintaining the cell’s redox balance and in the protection against reactive oxygen species (ROS). ROS plays a significant role in stem cell proliferation and migration [31]. It was demonstrated that short-term repopulating stem cells express higher levels of ROS than quiescent long-term repopulating stem cells. Low levels of ROS are essential for maintaining stem cell self-renewal. Whereas, high ROS levels, due to stress and inflammation, induce stem cell differentiation and enhanced their motility and mobilization [31]. Interestingly, it was recently shown that reducing oxygen stress enhances aged mouse hematopoietic stem cell numbers and function [32]. Previous studies have shown that increased oxidative stress induces FasL expression by T-lymphocytes [33], microglial cells [34], and endothelial cells [35, 36]. Over expression of FasL/FasR during inflammation is thought to negatively control immunity.

In our previous paper we showed that change in graft composition reduces levels of inflammation and promotes HSCs engraftment and hematopoietic recovery in the NSG mice model [9]. Our novel pre-clinical and clinical results further support these findings. Our results suggest that FasL may induce self-renewal potency of the graft by directly affecting CD34^+^ stemness. Highly relevant to our latest results, we had previously shown that FasL treatment of MSCs promote higher proliferation and stemness of progenitor cells while preserving wide differentiation potential and enhances immunomodulatory and immunosuppression activity [37]. In our recent open-label phase 1/2 study (ClinicalTrials.gov Identifiers: NCT02828878) FasL-treated MPBCs grafts (ApoGraft) demonstrate safety and preliminary efficacy in prevention of acute GvHD in match related BMT recipients. Good engraftment was also observed. Neutrophil and platelet engraftment was observed on day 14 in all treated patients [38]. Engraftment at relatively early-stage (compared to historical data [39–42]) may be the result of the above reported positive effect of FasL on CD34^+^ cells. More specifically, it may be a result of the positive effect of FasL on CD34^+^ cell differentiation into MkPs. It was previously shown that increasing the number of megakaryocytic progenitor cells in stem cell transplants by ex vivo expansion culture may provide an approach to accelerate platelet engraftment after high-dose chemotherapy [43–48]. These results are of clinical significance since time to engraftment is the most important variable for a better overall survival after stem cell transplant [49].

In this study, we present for the first time that FasL treatment of immune cells increased their “eat me” signals and led to their phagocytosis by activated macrophages. Phagocytosis of immune cells by activated macrophages reduced the ability of activated macrophages to secrete pro-inflammatory cytokines such as IL-6, TNF-α and IL-8. Improved Macrophage activity is considered as a tolerogenic effect [50]. Most importantly, in correlation with our in vitro data, the ex vivo treatment of MPBCs with FasL prior to transplantation in conditioned NOD-SCID IL-2Rγnull (NSG) mice prevented GvHD and improved stem cell transplantation.

FasL treatment of MPBCs has also the potential to suppress cellular immune responses via regulatory T-cells (Tregs). It was shown that APCs that have picked up apoptotic T-cells interact in the spleen with cells of the same antigen specific lineage. This leads to a suppressive effect on cellular immunity by the production of regulatory T-regs. These T-regs are also antigen-specific, meaning they correspond to the same clones of cytotoxic T-cells that were present in the apoptotic cell product [51]. The FasL AM treated MPBCs product may therefore reduce the immunity of the graft, by depleting donor immune cells. Cell depletion from donor grafts has been used to prevent GvHD. Positive selection of the CD3^+^ population, which can reduce response to self-antigens and reduce the donor and recipient immune system reactivity by inhibiting antigen presentation due to overloading of the macrophages by apoptotic cells of the graft [28] and by production of antigen specific T-regs.

During stem cell engraftment, donor T cells interact with both host and donor APCs; host APCs present host major and minor histocompatibility antigens to donor T cells priming the acute GvHD reaction [6].

Ex vivo and In vivo hematopoietic stem cell positive selection or enrichment, and depletion of target T cell populations are strategies for GvHD prevention [7]. Depletion of GvHD causing cells by administration of cyclophosphamide amide 3 days post-transplantation is used primarily in haploidentical transplants [52]. These modalities can have deleterious side-effects, which may include delayed immune and attenuation of GvT effects.

There is an ongoing need in cell therapy manufacturing to keep unpicking the complexity and variation sources (biological, technical and operator), and the consequences of these variations, and how to address/control them. Manufacturing experience has shown that variability can be reduced by automation of manual processes [53].

The current manufacturing processes for cell-based therapeutics are largely manual and complex. These are highly laborious, often involving open processes which are difficult to scale-up and rely heavily on the operator’s experience and judgment and inherently pose sterility and uniformity challenges. Consequently, these manufacturing processes are prone to human error and can result in increased batch-to-batch variability, high manufacturing costs, increased risk of contamination and batch loss. Automation can provide more control over a bioprocess using sensors that produce online and continuous measurements, while leading to a more accurate and faster process optimization. Besides biological variation, which is difficult to tackle due to the complexity of these products, in-process variation occurring from human handling is another persistent challenge that can impact product quality [54]. FasL automated treatment of MPBCs significantly reduce manufacturing time to ~4 h and the number of operators to one, all under aseptic conditions. It increases manufacturing efficiency and robustness and reduces the risk of product contamination and variability – all without compromising product quality. These direct effects are also expected to reduce QC, QA and regulatory burdens (e.g., taking manufacturing out of the clean room) and consequently reduce the cost of production.

HSCT is a very robust and powerful tool that has been used for over 60 years. Unfortunately, it carries significant adverse effects, mostly related to the differences between the donor and recipient’s immune systems with the main manifestation being GvHD. The modalities currently used for prevention and treatment of aGvHD are significantly jeopardized by reduction of the graft’s effectiveness (engraftment and persistent chimerism, GvT), increase in recurrency of the underlying disease and the morbidity associated with the treatment itself. The transplant community has long been vexed by a therapeutic dilemma regarding GvHD prophylaxis for patients suffering from malignant diseases – “too little” prophylaxis can result in life-threatening GvHD, while “too much” prophylaxis may impair the GvT reactions and increase the risk of disease relapse. Therefore, HSCT use has been restricted mainly in life-threatening cases where the benefit outweighs the risk, mainly in hematological malignancies.

From around the 90’s, as HSCT protocols and GvHD prophylaxis improved, there has been growing attempts to expand the use of HSCT to other non-malignant and immune-related indications [55].

The purpose of HSCT in autoimmune diseases (ADs) and immune-related conditions is to reduce/eliminate the autoreactive immune cells and either reset the immune system in the case of autologous HSCT or replace it in the case of allogeneic HSCT.

Although autologous HSCT is safer and carries less complications and mortality compared to allogeneic HSCT, it is less potent in cases such as refractory ADs, where replacement of the patient’s immune system has higher chances of autoreactive cells eradication [55–57].

HSCT for autoimmune diseases is currently used in Multiple sclerosis (MS), connective tissue diseases such as Systemic sclerosis (SSC) and Systemic lupus erythematosus, Rheumatoid arthritis, Inflammatory bowel diseases, Hematologic diseases such as Immune thrombocytopenia, Vasculitis, neurological diseases, Insulin dependent diabetes and other autoimmune and monogenic diseases [56, 58, 59].

FasL AM treatment of MPBCs can be used for either resetting or replacing the immune system in ADs, potentially attenuating HSCT associated toxicity while maintaining graft’s effectiveness, thus widening its use for these indications.

The use of immunosuppressants (IS) to prevent organ rejection and GvHD in solid organ transplant patients is associated with nephrotoxicity, increased risk of opportunistic infections, cardiovascular, metabolic effects, and cancer [60].

Various strategies have been employed to reduce the negative impact of chronic IS and improve quality of life and overall outcome, however, success has been erratic at best. Therapeutic agents that have been developed to avoid the side effects of IS also have not lived up to their promises. They too are associated with their own set of complications [60]. Induction of allograft tolerance has been considered the logical solution for many of these limitations associated with long-term IS as well as the best approach to reduce the risk of chronic rejection [60]. One strategy to eliminate the use of IS is by establishing donor chimerism in recipients by the transplantation of donor-derived hematopoietic cells [61].

There is a considerably greater risk of GvHD in stable complete chimerism, as compared with stable mixed chimerism [60]. Thus, for purposes of tolerance induction to organs, defined as the specific absence of a destructive immunologic response to a transplanted organ or tissue without ongoing exogenous IS, the achievement of stable mixed chimerism instead of complete chimerism is more desirable [60, 62].

However, to-date, mixed chimerism strategies to induce complete IS freedom were only successful in matched-related HSCT [60]. This, might be because of the cell-selection and depletion technologies used, leading to loss of donor chimerism and allograft tolerance in mismatched HSCT transplant patients. We hypothesize that FasL AM treatment of MPBCs will induce sustained chimerism, with lower incidence and severity of GvHD risk, thereby allowing tolerance and complete IS freedom in HLA-mismatched renal transplant recipients.

Sepsis and ARDS are life threating conditions with high mortality and morbidity. After decades of research, and numerous pre-clinical and clinical trials, sepsis and ARDS remain a critically unmet need, and essentially, the management remains supportive. During the last few years, cell therapies have shown therapeutic potential for ARDS and sepsis. Cellular treatment through BMT have also been tested but while autologous BMT did not show therapeutic effects, the allogeneic BMT was challenged by the GvHD toxicity [63].

A number of studies and trials have explored the role of extracorporeal photopheresis (ECP) in the treatment of scleroderma, GvHD and other autoimmune conditions. Despite the numerous therapies used for patients with SSc, disease-related morbidity and mortality remain high [64]. As MPBCs apheresis product contain fewer than 3% mobilized stem cells [1] we postulate that FasL treatment of non-mobilized peripheral blood cells (PBCs) apheresis product will have a selective cell elimination effect on mature and activated immune cells. Indeed, FasL-incubated PBCs show a similar reduction in activated immune cells as FasL-incubated MPBCs (data not shown).

Taking this into consideration, our FasL functional selection technology works on blood apheresis products by reducing the graft’s alloreactivity while preserving and probably improving its effectiveness. This technology can potentially be used on various cell sources including mobilized apheresis products for reset or replacement of a dysfunctional immune system, in the case of autologous or allogeneic BMT, or using of non-mobilized apheresis for immune system optimization. Furthermore, its use is not limited to GvHD prevention in hematological malignancies and can also be used to treat other hematological disorders, autoimmunity, as well as immune-related conditions such as immune rejection (e.g., GvHD and solid organs rejection), ARDS, CRS, CAR-T-related and COVID-19 cytokine storm etc.

## Supplementary information

Supplementary Tables

Legends to Supplementary figures

Supplementary Figure 1

Supplementary Figure 2

Supplementary Figure 3

Supplementary Figure 4
