# Supplementary material for: Short treatment of peripheral blood cells product with Fas ligand using closed automated cell processing system significantly reduces immune cell reactivity of the graft in vitro and in vivo
Source: Bone Marrow Transplant. 2022 May 10;57(8):1250–9. doi: 10.1038/s41409-022-01698-3 (PMC9088133; doi:10.1038/s41409-022-01698-3)
Supplement: Supplementary file 1 — Supplementary Tables [file 41409_2022_1698_MOESM1_ESM.docx]

**Supplementary Table 1. The absolute number of total nucleated cells, CD34+, and percent of immune subsets before and after treatment**

| **Sample** | **Parameter** | **1** | **2** | **3** | **4*** | **5** | **6** | **7** | **8** | **9** | **10** | **11** | **12**** | **13** | **14** | **15** | **16** | **17** | **18** | **19** | **20** |
| --- | --- | --- | --- | --- | --- | --- | --- | --- | --- | --- | --- | --- | --- | --- | --- | --- | --- | --- | --- | --- | --- |
|  | FasL dose for treatment (ng/ml) | 100 | 100 | 100 | 100 | 100 | 100 | 100 | 100 | 100 | 100 | 100 | 100 | 100 | 100 | 100 | 100 | 100 | 400 | 400 | 400 |
| MPBCs | TNC x10^6^ for treatment | 3818 | 2879 | 4848 | 4193 | 20318 | 3840 | 4784 | 4428 | 26820 | 3180 | 4932 | 3705 | 2429 | 3692 | 45934 | 105225 | 4121 | 3102 | 3432 | 3245 |
|  | CD34^+^ x10^6^ for treatment | 28 | 25 | 27 | 35 | 219 | 61 | 57 | 42 | 362 | 42 | 39 | 66 | 49 | 41 | 193 | 705 | 86 | 30 | 22 | 57 |
|  | CD45^+^ (% of analyzed cells) | 99.4 | 98.7 | 96.1 | 98.9 | 99.1 | 98.8 | 97.0 | 96.8 | 98.7 | 94.2 | 97.9 | 98.6 | 98.4 | 98.5 | 94.8 | 98.4 | 98.0 | 99.3 | 97.7 | 97.5 |
|  | CD3^+^ (% of CD45^+^) | 48.0 | 21.2 | 52.5 | 33.7 | 52.9 | 36.8 | 37.3 | 46.3 | 48.1 | 26.8 | 38.9 | 35.1 | 17.5 | 42.9 | 58.5 | 43.0 | 25.4 | 31.1 | 30.8 | 33.7 |
|  | CD34^+^ (% of CD45^+^) | 0.74 | 0.85 | 0.55 | 0.83 | 1.08 | 1.58 | 1.19 | 0.96 | 1.35 | 1.31 | 0.80 | 1.79 | 2.01 | 1.11 | 0.42 | 0.67 | 2.1 | 0.98 | 0.63 | 1.77 |
|  | CD38Neg/Low (% of CD34+ cells) | - | - | - | - | - | 20.7 | 14.0 | 8.2 | 13.3 | 8.3 | 15.0 | 11.1 | - | - | 14.5 | 19.2 | - | 4.6 | 11.2 | 3.4 |
|  | CD25^+^ (% of CD3^+^) | - | - | 19.6 | 11.6 | 14.9 | - | 14.7 | - | - | 13.5 | 8.7 | 16.9 | - | - | - | - | - | - | - | - |
|  | CD33^+^ (% of CD45^+^) | - | - | 12.2 | 23.2 | 27.6 | - | 22.6 | - | - | 35.7 | 35.6 | 29.9 | - | - | - | - | - | - | - | - |
|  | CD33^+^ HLA-DR high (% of CD33^+^) | - | - | 15.6 | 13.0 | 24.1 | - | 18.7 | - | - | 15.8 | 16.0 | 21.2 | - | - | - | - | - | - | - | - |
|  | CD19^+^ (% of CD45^+^) | - | - | 9.8 | 8.8 | 11.0 | - | 10.2 | - | - | 3.3 | 3.0 | 2.8 | - | - | - | - | - | - | - | - |
|  | CD19^+^ HLA-DR high (% of CD19^+^) | - | - | 7.5 | 5.6 | 5.1 | - | 12.1 | - | - | 17.7 | 20.5 | 4.9 | - | - | - | - | - | - | - | - |
| MPBCs  +FasL AM | TNC x10^6^ after treatment | 4037 | 1999 | 2954 | 3300 | 12539 | 3271 | 3176 | 2828 | 21983 | 2002 | 3575 | 3052 | 1722 | 3136 | 35385 | 65550 | 2943 | 2248 | 2139 | 2438 |
|  | CD34^+^ x10^6^ after treatment | 31 | 16 | 18 | 36 | 228 | 49 | 46 | 39 | 320 | 28 | 33 | 50 | 30 | 40 | 160 | 551 | 53 | 23 | 17 | 47 |
|  | CD45^+^ (% of analyzed cells) | 99.6 | 99.5 | 99.1 | 99.8 | 99.1 | 99.6 | 99.5 | 98.9 | 97.3 | 99.4 | 99.4 | 99.0 | 99.1 | 99.6 | 94.9 | 97.4 | 99.6 | 99.4 | 95.6 | 96.9 |
|  | CD3^+^ (% of CD45^+^) | 50.4 | 19.9 | 45.4 | 29.2 | 19.2 | 42.6 | 24.7 | 40.3 | 39.3 | 27.5 | 29.5 | 26.8 | 18.5 | 34.2 | 58.8 | 29.6 | 24.2 | 34.9 | 36.8 | 22.4 |
|  | CD34^+^ (% of CD45^+^) | 0.76 | 0.81 | 0.6 | 1.10 | 1.82 | 1.51 | 1.45 | 1.37 | 1.46 | 1.42 | 0.93 | 1.65 | 1.72 | 1.27 | 0.5 | 0.84 | 1.8 | 1.01 | 0.80 | 1.95 |
|  | CD38^Neg/Low^ (% of CD34^+^ cells) | - | - | - | - | - | 13.9 | 14.4 | 8.1 | 14.1 | 8.8 | 15.5 | 10.3 | - | - | 13.2 | 16.6 | - | 5.5 | 12.5 | 4.0 |
|  | CD25^+^ (% of CD3^+^) | - | - | 15.0 | 6.6 | 8.4 | - | 9.7 | - | - | 7.4 | 5.0 | 10.4 | - | - | - | - | - | - | - | - |
|  | CD33^+^ (% of CD45^+^) | - | - | 6.5 | 21.9 | 20.5 | - | 24.8 | - | - | 16.4 | 16.6 | 22.0 | - | - | - | - | - | - | - | - |
|  | CD33^+^ HLA-DR ^high^(% of CD33^+^) | - | - | 12.3 | 6.6 | 12.5 | - | 15.9 | - | - | 11.2 | 10.4 | 16.8 | - | - | - | - | - | - | - | - |
|  | CD19^+^ (% of CD45^+^) | - | - | 8.0 | 4.7 | 7.0 | - | 6.0 | - | - | 3.1 | 3.4 | 2.3 | - | - | - | - | - | - | - | - |
|  | CD19^+^ HLA-DR ^high^(% of CD19^+^) | - | - | 4.3 | 2.1 | 3.6 | - | 7.8 | - | - | 8.1 | 12.3 | 1.6 | - | - | - | - | - | - | - | - |

*This sample was used for GvHD evaluation in vivo experiment

**This sample was used for GvHD evaluation and engraftment in vivo experiments

**Supplementary Table 2. Cell number administered to mice in in vivo experiments**

| **Experiment No** | **Test mode** | **Test item** | **Administrated Cells x 10^6^ /mouse** | | | | | |
| --- | --- | --- | --- | --- | --- | --- | --- | --- |
|  |  |  | **TNC** | **CD34^+^** | **CD3^+^** | **CD25^+^** | **CD33^+^** | **CD19^+^** |
| 1 (Figure 5 A and B) | GvHD | MPBCs | 5 | 0.04 | 1.7 | 0.6 | 1.2 | 0.4 |
|  |  | MPBCs+FasL AM | 5 | 0.06 | 1.5 | 0.3 | 1.1 | 0.2 |
| 2 (Figure 5 C and D) | GvHD | MPBCs | 4.5 | 0.08 | 1.6 | 0.8 | 1.3 | 0.1 |
|  |  | MPBCs+FasL AM | 4.5 | 0.08 | 1.2 | 0.5 | 1.0 | 0.1 |
| 2 (Figure 5 E-H) | Engraftment | MPBCs | 3 | 0.05 | 1.1 | 0.5 | 0.9 | 0.1 |
|  |  | MPBCs+FasL AM | 3 | 0.05 | 0.8 | 0.3 | 0.7 | 0.1 |
