# Supplementary material for: Short treatment of peripheral blood cells product with Fas ligand using closed automated cell processing system significantly reduces immune cell reactivity of the graft in vitro and in vivo
Source: Bone Marrow Transplant. 2022 May 10;57(8):1250–9. doi: 10.1038/s41409-022-01698-3 (PMC9088133; doi:10.1038/s41409-022-01698-3)
Supplement: Supplementary file 2 — Legends to Supplementary figures [file 41409_2022_1698_MOESM2_ESM.docx]

**Supplementary Figure 1. FasL AM treatment effect on major cell populations of MPBCs**

(A)Lymphocytes, granulocytes, and monocytes of tested MPBCs and FasL AM treated MPBCs were gated according to SSC and percent of each population was analyzed out of CD45^+^ cells

FasL AM treatment significantly reduces the percentage of lymphocytes (B) and, accordingly, increases the percentage of granulocytes (C), monocytes are not affected (D).

N=17. Mean ±SEM, *P ≤0.05, *** P ≤0.001; Paired T-test

**Supplementary Figure 2. Gating Strategy of Phagocytic macrophages FACS analysis**

CD11b positive MPBCs were excluded from analysis based on Macrophages only and MPBCs only stained samples.

**Supplementary Figure 3. T cell activation and T regulatory cells composition in FasL treated MPBCs**

The percentage of CD25 expressing T helper (A) and T cytotoxic (B) cells significantly decrease in FasL treated MPBCs, compared to MPBC control cells, but percent of regulatory, CD127^+low^, T cells significantly increased (C). The ratio of Tregs/non-Tregs (out of total activated cells) is significantly changed, resulting in elevation of the Tregs fraction (D), suggesting that the FasL treated MPBCs composition is drawn towards more anti-inflammatory reaction in comparison to untreated MPBCs. N=11; Mean +SEM, *P<0.05, **P<0.01, ****P<0.0001; Pared T-test

**Supplementary Figure 4.** **FasL treatment does not affect the ability of activated T cells to proliferate and kill leukemic cells**

FasL (100ng/ml) treated and untreated expanded T cell (from 12 days in culture) were added in different ratio of leukemic U937 and MV4-11 cells to T cells; 1:1, 1:5, 1:10, 1:30, (2x10^4^ /2x10^4^, 10x10^4^, 20x10^4^ 60x10^4^ in additional 100µl medium). No difference in the ability of untreated (T cells from MPBCs), treated with incubation medium (T cells from MPBCs+FasL) or treated with 100ng/ml FasL (T cells from MPBCs+FasL) expended cells to eradicate U937 (A) or MV4-11 (B) leukemic cells was shown.
