# Supplementary figures and images for: Short treatment of peripheral blood cells product with Fas ligand using closed automated cell processing system significantly reduces immune cell reactivity of the graft in vitro and in vivo
Source: Bone Marrow Transplant. 2022 May 10;57(8):1250–9. doi: 10.1038/s41409-022-01698-3 (PMC9088133; doi:10.1038/s41409-022-01698-3)

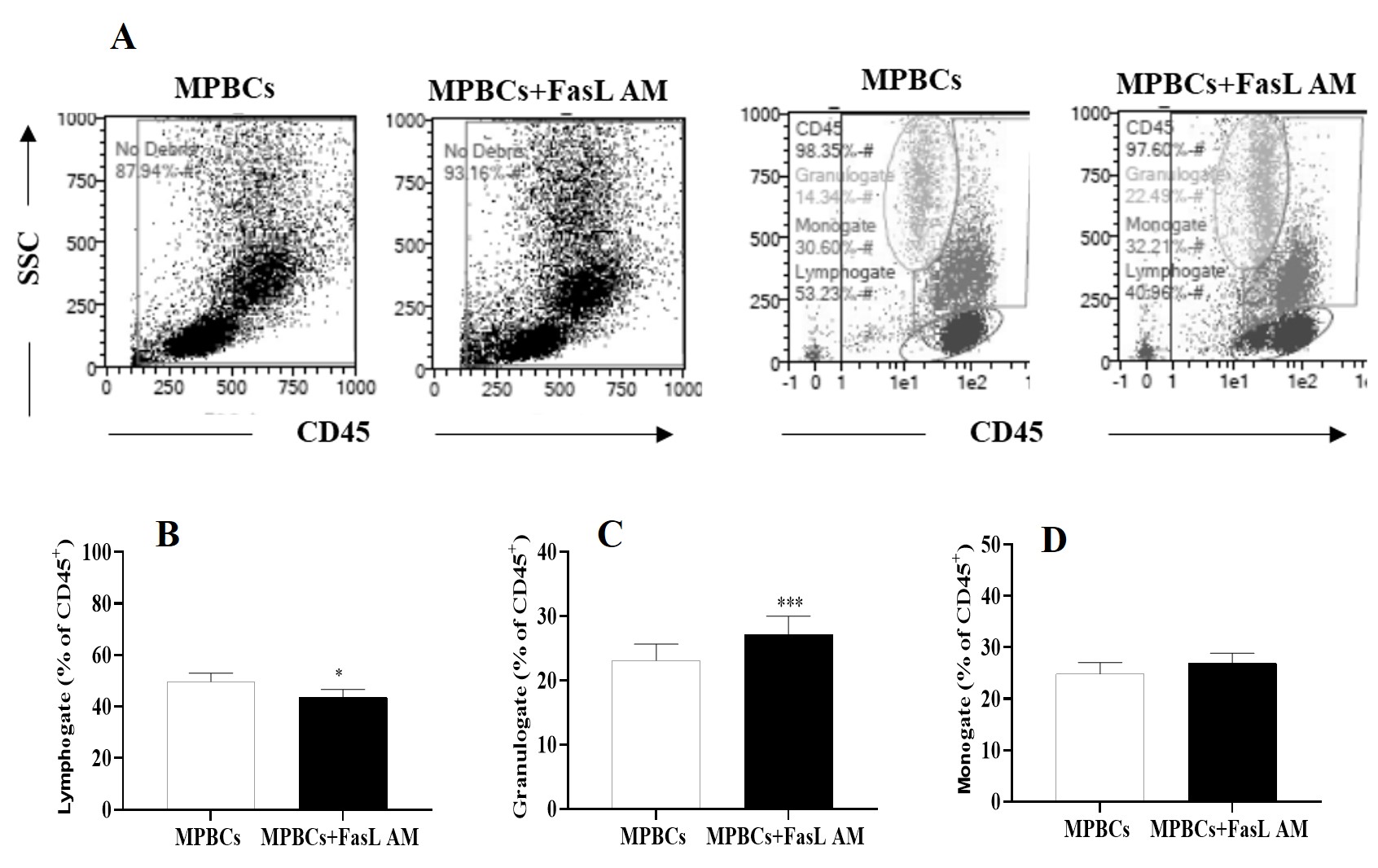

Supplement: Supplementary file 3 — Supplementary Figure 1 [file 41409_2022_1698_MOESM3_ESM.jpg]

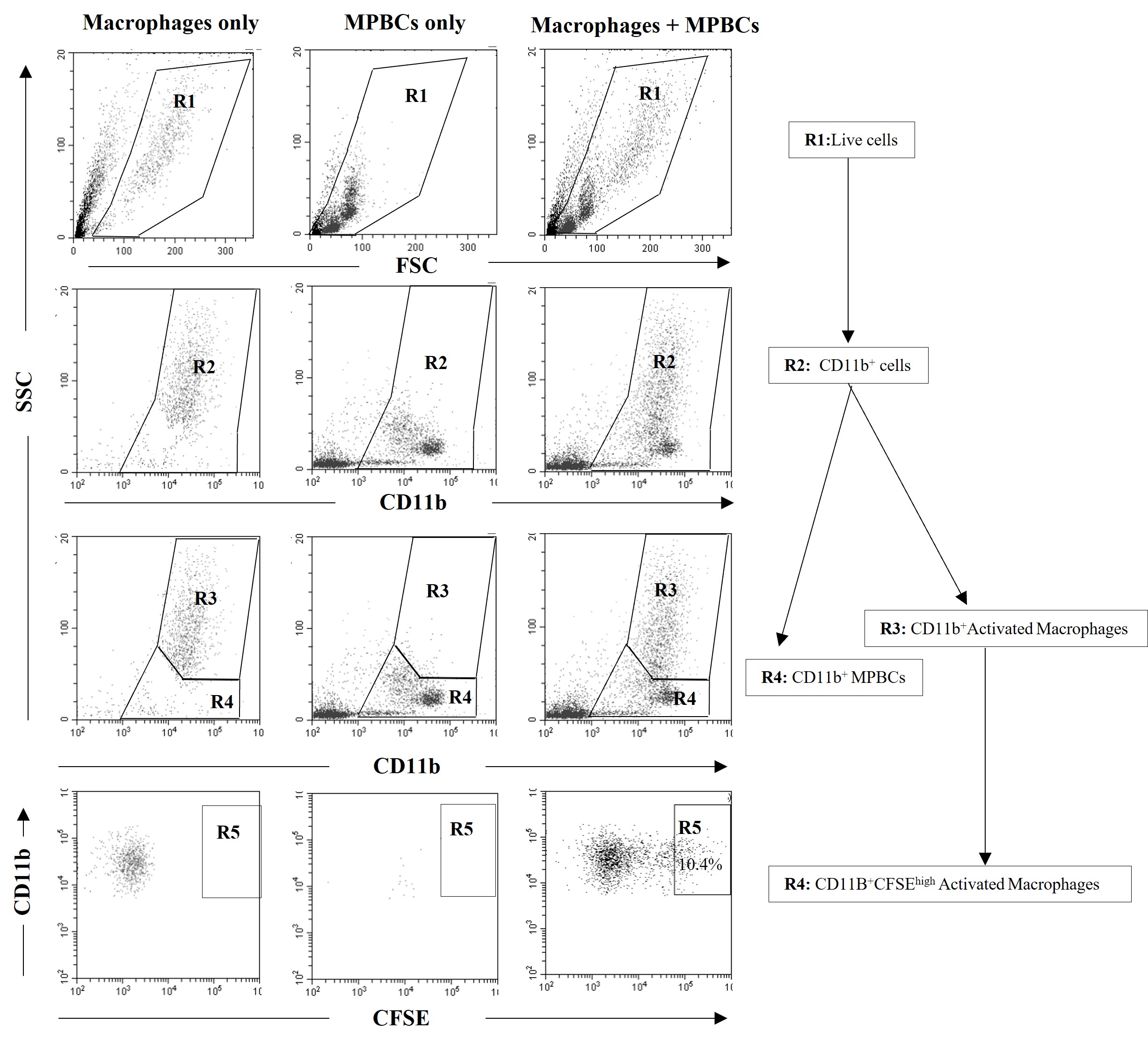

Supplement: Supplementary file 4 — Supplementary Figure 2 [file 41409_2022_1698_MOESM4_ESM.jpg]

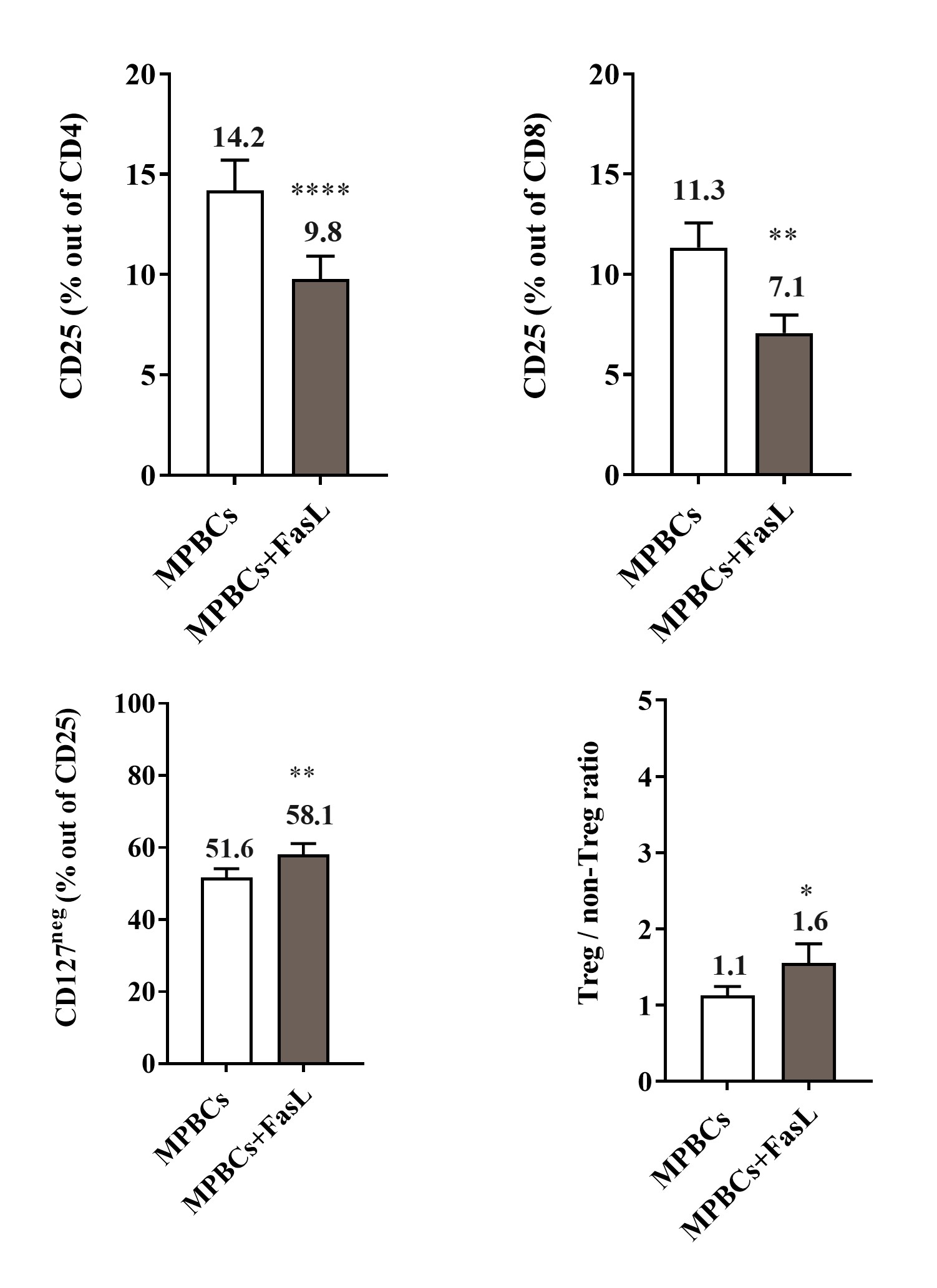

Supplement: Supplementary file 5 — Supplementary Figure 3 [file 41409_2022_1698_MOESM5_ESM.jpg]

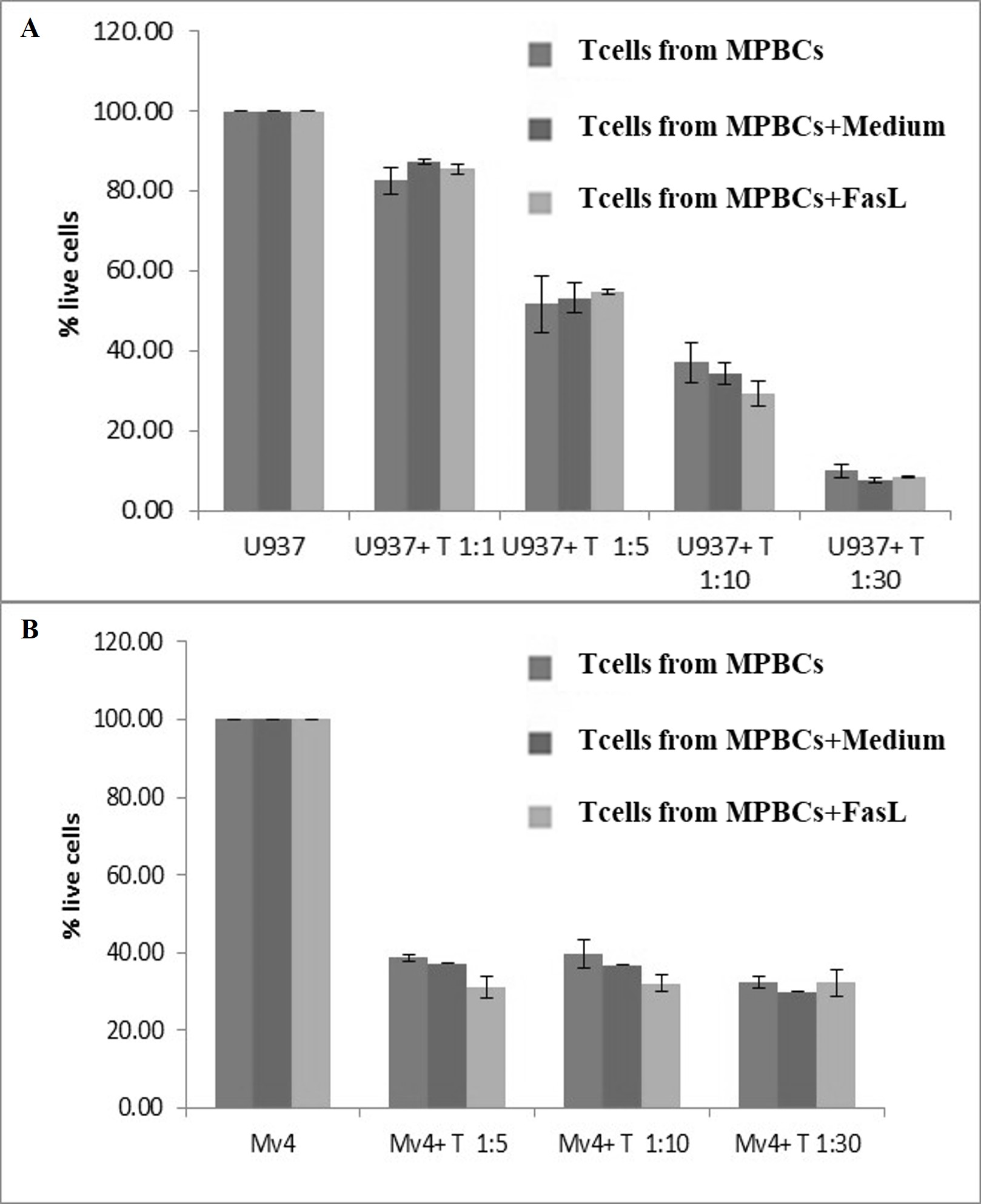

Supplement: Supplementary file 6 — Supplementary Figure 4 [file 41409_2022_1698_MOESM6_ESM.jpg]
